# Modelling key ecological factors influencing the distribution and content of silymarin antioxidant in *Silybum marianum* L.

**DOI:** 10.1371/journal.pone.0322442

**Published:** 2025-07-11

**Authors:** Mahboobe Hojati, Ruhollah Naderi, Mohsen Edalat, Hamid Reza Pourghasemi

**Affiliations:** 1 Department of Plant Production and Genetics, School of Agriculture, Shiraz University, Shiraz, Iran; 2 Department of Soil Science, School of Agriculture, Shiraz University, Shiraz, Iran; Quaid-i-Azam University Islamabad: Quaid-i-Azam University, PAKISTAN

## Abstract

The increasing demand for natural medicine has increased the significance of *Silybum marianum* as a valuable medicinal plant. It is used to restore liver cells; reduce blood cholesterol; prevent prostate, skin, and breast cancer; and protect cervical cells and kidneys. To identify ecological factors affecting the distribution and amount of silymarin in *S. marianum* three machine learning algorithms including boosted regression trees (BRT), random forest (RF), and support vector machines (SVM) have been applied in Fars Province, Iran. Fourteen factors affecting *S. marianum* growth and development were determined and subsequently converted into raster maps for the modeling phase using a Geographic Information System (GIS). Subsequently, the Receiver Operating Characteristic (ROC) curve and random forest algorithm were used to evaluate the models and the significance of the factors, respectively. Results showed that The RF (ROC: 0.99), BRT (ROC: 0.98), and SVM (ROC: 0.96) models were highly accurate in predicting the habitat suitability of *S. marianum*. The results of the RF algorithm also revealed that factors such as distance from roads, elevation, and mean annual rainfall had the most significant influence on the habitat suitability of *S. marianum*. In addition, the mean annual rainfall, mean annual temperature, and elevation had the highest effects on silymarin accumulation. In general, the northern and northwestern regions of the Fars Province offer optimal environmental conditions for the growth of *S*. *marianum*. The southern and southwestern regions of Fars Province, characterized by higher temperatures and lower precipitation, are suitable for the enhanced biosynthesis of silymarin and expansion of its cultivation and production. This study provides a robust framework for understanding the ecological preferences of *S*. *marianum* and optimizing its cultivation and management for pharmaceutical applications. By identifying the most influential environmental variables, this research has the potential for the sustainable utilization of this species, enhancing both its conservation and use as a medicinal resource.

## 1. Introduction

Milk thistle (*Silybum marianum* L.) is one of the most important plants in the Asteraceae family, and can be both an annual and biennial species [1]. It is native to southern Europe, Mediterranean, and North Africa. It is also used as a medicinal plant in Australia, New Zealand, and North and South America [2,3]. According to Shokrpour et al. [4], *S. marianum* ecotypes are endangered because of overgrazing, poor farm management, and increasing pastures across several geographical regions of Iran, particularly in the north, northwest, west, and southwest. This herb has been used since ancient years to cure chronic liver illnesses and to protect the liver from toxins. It has been known for millennia as a liver booster [5]. *Silybum marianum* can be utilized to produce polymers or biodiesel, and can also be used as animal feed and seed oil [6,7].

The therapeutic properties of *S*. *marianum* have been attributed to the concentration of active flavonolignans, with silymarin being one of the most significant components [8,9]. Silymarin, a complex mixture of flavonolignans including silybin, silydianin, and silychristin, has been acknowledged for its numerous applications in agriculture as well as its therapeutic potential in human medicine [10,11]. Silymarin is a strong antioxidant in *S. marianum*. It is used to restore liver cells, reduce blood cholesterol, prevent prostate, skin, and breast cancer, and protect cervical cells and kidneys [12].

In recent years, the market for medicinal herbs and nutritional supplements has experienced substantial growth, with this product which now ranking among the top-selling medicinal herbs in several countries, including the US and Italy [3,13]. Some wild species of medicinal plants are being overexploited due to the increasing demand [14,15]. Thus, it is necessary to cultivate them extensively under conditions conducive to enhancing the quality of their beneficial components [16].

The distribution of medicinal plants from one location to another was determined based on the relationship between plants and their growth conditions [17]. Various ecological factors such as mean annual rainfall, mean annual temperature, and minimum temperature during the coldest season of the year significantly affect the geographical distribution of species [18]. Understanding this relationship is vital because environmental and climatic factors play pivotal roles in determining the success or failure of medicinal plant cultivation [19,20]. Models play a crucial role in predicting plant ecological processes, offering insights that enhance our understanding of agricultural systems and enable informed decision-making essential to agricultural engineering [21]. In recent years, the integration of artificial intelligence (AI) and GIS into agricultural models has attracted significant interest from researchers. GIS has made significant changes in modern/precision agriculture by designing quality maps and specifying the spatial and temporal changes in plant and soil characteristics [22].

Species distribution models (SDM) use field-measured data, supplementary maps, and specialist knowledge to quantify the connection between the spread of plants and environmental factors and to estimate the real or potential distribution of a species [23]. SDM assesses the relationship between environmental conditions and plant dispersion to determine its potential range. These models can predict current and future species distributions, quantify the impacts of environmental factors, and estimate their distribution in data-scarce regions [24]. Critical aspects of this application require careful selection of appropriate habitats to guarantee the accuracy and reliability of predictions and access to relevant high-quality data.

At the regional scale, including Fars Province in Iran, *S. marianum* is of ecological and economic importance because of its medicinal value and potential as a source of income for local communities. However, understanding its distribution and predicting suitable habitats is critical for sustainable harvesting and prevention of potential invasiveness. Previous studies have demonstrated the utility of SDM based on machine learning techniques, such as RF, BRT, and SVM, to accurately predict plant species habitats [25–27]. These models have proven to be particularly effective when integrated with GIS and remote sensing data.

Globally, studies such as those by Mollalo et al. [28] and Kohansarbaz et al. [29] have highlighted the relative performance of machine-learning algorithms in mapping vegetation. For instance, RF consistently outperformed SVM and BRT in accuracy metrics, whereas other approaches, such as “artificial neural networks (ANN)” and “gradient boosting machines (GBM)”, also demonstrated promise under certain conditions. Incorporating these insights at the regional level can enhance predictions for *S. marianum* in “Fars Province”, considering the unique environmental and climatic characteristics of the area.

This study employed the RF, SVM, and BRT models with the utmost confidence to accurately pinpoint the key ecological features and regions where *S. marianum* is highly likely to exist. In addition, Used the RF model to identify the most crucial ecological parameters influencing the active ingredients of silymarin. *S. marianum* is an extremely valuable therapeutic plant that can be grown in the Fars Province. This plant can be cultivated by creating maps that identify the most active, ent-rich, and vulnerable areas. This plant is one of Iran’s most significant medicinal plants, and plays a crucial role in preventing ecosystem destruction resulting from the region’s reliance on wild plants. Although many studies worldwide have examined the bioactivity, phytochemistry, and genetics of milk thistle, there has been relatively little exploration of its basic agronomy and farming potential. In this context, the current study aimed to determine the performance of the RF, BRT, and SVM models on the distribution of *S*. *marianum* growth and silymarin production in Fars Province. The findings of this study can be used as a decision-making tool by farmers and researchers to optimize the cultivation, development, and industrial production of this medicinal plant to increase yield and product quality. Also, these findings can help develop new strategies for the industrial production of plant active ingredients and the development of medicinal plant cultivation. This research specifically addressed the following questions: 1) How do different ecological factors influence the habitat suitability of *S. marianum* in different regions of Fars Province? (2) What are the most significant environmental variables affecting silymarin accumulation in *S. marianum* across various climatic regions?

## 2. Materials and methods

**Fig 1:**
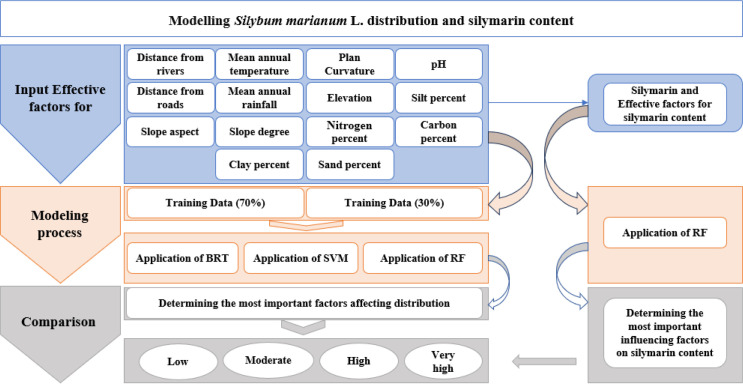
The flowchart of the modeling process of *S. marianum* habitat and its production.

Fig 1 illustrates the methodological flowchart of this study, encompassing inventory mapping, the division of data into training and validation sets, the selection and preparation of environmental predictors, the modeling procedure, the validation of the outcomes, and the selection of the superior model.

### 2.1. Study area

**Fig 2:**
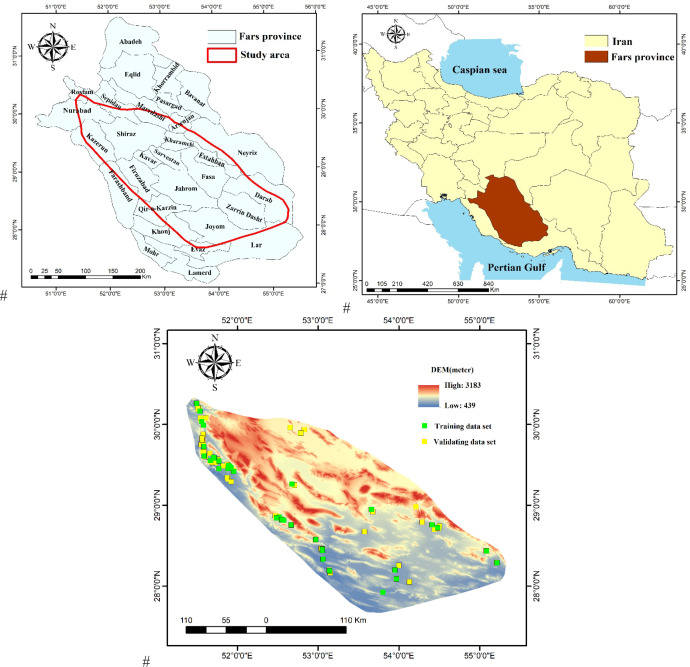
The research area’s location in Iran’s Fars province.

This study was conducted in southern Iran’s Fars Province (Fig 2), which extends between 27.05° and 31.67° (N) and between 50.60° and 55.58° (E). This province covers an area of approximately 12.4 million ha, of which pastures represent the 86.29% [30]. The region experiences a varied climate, characterized by an average annual rainfall of 300 mm, an average annual temperature of 17 °C, and an average elevation of 1500 m [31], and supports a rich variety of vegetation, including 144 valuable plant species [32]. However, overgrazing, harvesting of plant species, and land use change have led to the destruction of rangelands and the decline of natural plant species in this province over the past decade [33,34].

### 2.2. Methodology

#### 2.2.1. Mapping of *S. marianum* presence.

**Fig 3:**
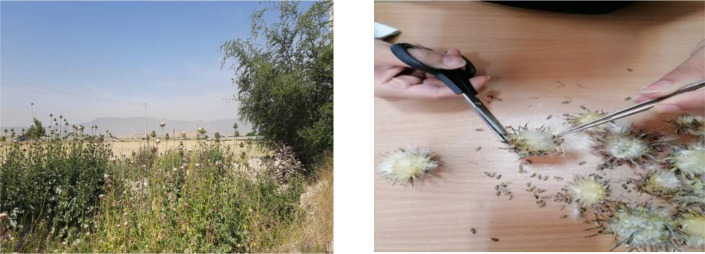
Identification and collection of *S. marianum* in the study area (Taken photo by SX710 HS Canon Camera, Mahbobe Hojati).

A total of 117 *S. marianum* sites were found in all counties of the Fars Province during a field study conducted in 2021. These locations were recorded using a “global positioning system (GPS)” device (Garmin Map 62s, USA) (Fig 3). Soil and seed samples were collected during the physiological ripening phase at the location where the medicinal plant is found. Ecological factors such as the slope degree, elevation, slope aspect, plan curvature, clay percentage, sand percentage, silt percentage, carbon percentage, pH, nitrogen percentage, mean annual rainfall, mean annual temperature, distance from rivers, and distance from roads, were used for determining their correlation with species distribution and the active component silymarin [35,36]. In the current study, 70% of the presence data (82 locations) were used to train the models and the remaining 30% were used for the validation phase [37].

### 2.3. Preparation of variables

**Fig 4:**
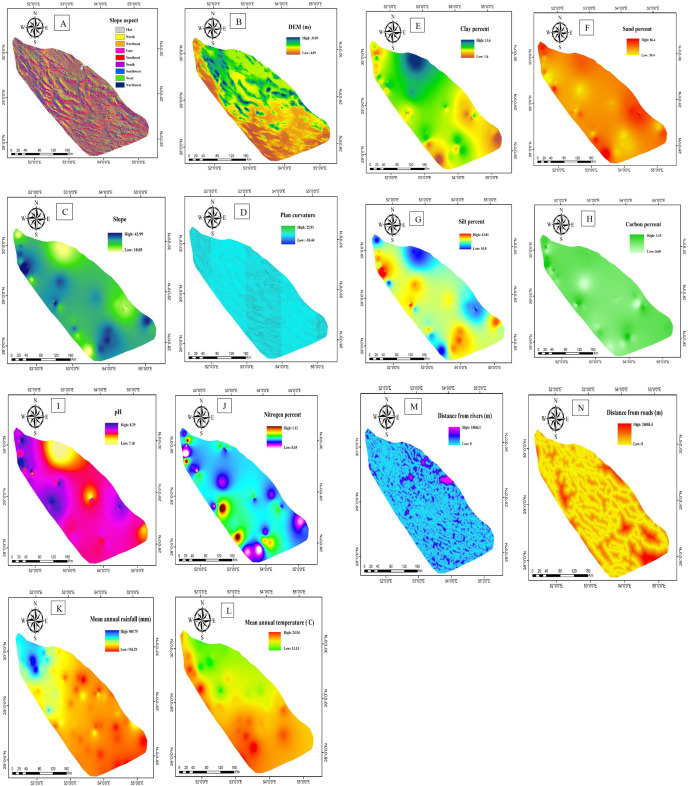
Effective factors on the growth and development of *S. marianum*: “slope aspect” (A), “DEM (m)” (B), “slope angle” (C), “plan curvature” (D), “clay percent” (E), “sand percent” (F), “silt percent” (G), “carbon percent” (H), “pH” (I), “Nitrogen percent” (J), “mean annual rainfall (mm)” (K), “mean annual temperature (°C)” (L), “distance from rivers (m)” (M), and “distance from road (m)” (N).

The best sites for *S. marianum* habitats and their impact on the quantity of silymarin in Fars Province were modeled using a variety of ecological parameters. The beneficial topological variables were identified as elevation, slope, aspect, and plan curvature. To produce these parameters, a 30-meter-resolution Digital Elevation Model (DEM) map was utilized. Using ArcGIS 10.8.0 (http://data.aoos.org/maps/sensors/#l=sensor-stations), these factors were converted into raster layers (Fig 4A–4D).

The physical attributes of the soil (such as the proportions of sand, silt, and clay), chemical attributes (such as pH, EC, nitrogen, and organic matter), mean annual rainfall, mean annual temperature, and distance from roads and rivers were measured. Soil samples (117 sites) were collected from locations where *S. marianum* was found and processed at the Central Laboratory of Shiraz University, the Soil Science Laboratory, and the Azma Pars Laboratory.

To determine the physical characteristics of the soil (percentage of clay, sand, and silt), air-dried samples were first passed through a 2-mm sieve and then determined using the hydrometry method [38]. **The soil pH was determined using a pH meter** [39].

The amount of organic carbon (SOC) in the soil samples was measured using the Walkey-Block titration method and calculated using Equation 1 [40].


$$\begin{array}{c} \text{SOC}=\frac{(V\times N{)}_{k_{2}{cr}_{2}o_{7}}-(V\times N{)}_{Fe{\left({NH}_{4}\right)}_{2}({SO}_{4})}}{S\times 0.76}\times \frac{3}{1000}\times 100 \end{array}$$ (1)

where SOC is the weight of the dried soil (g); V_1_ and V_2_ are the ferrous ammonium sulfate volumes used in the control and the soil sample (mL), respectively; N is the ferrous ammonium sulfate normality; S is the dry weight of the soil sample in grams; and 0.003 is the conversion factor from the volume of titrant to grams of carbon, based on the relationship between the molecular weight and 0.76 is the portion of oxidized organic carbon, respectively.

The amount of nitrogen in the soil samples was determined by the Kjeldahl method based on Bremner’s instructions [41]. Parallel lines of temperature and precipitation were prepared by the Regional Meteorological Organization of Fars Province to create climatic layers. The average values of annual rainfall and temperature were calculated, and raster maps with a resolution of 30 m were created using the inverse distance weighting (IDW) algorithm [42] in “ArcGIS 10.8.0” (Fig 4K–4L). The smaller the distance of the points from the nearest cell, the more effective it is. Therefore, a set of points with different radus was used to create maps for better interpolation [43].

Similarly, vector maps of the road and river were used to prepare the study layers of distance from roads and rivers (scale of 1:25,000). Layers were created using the Euclidean distance (ED) algorithm in ArcGIS 10.8.0 (Fig 4M and 4N). This algorithm creates maps based on varying distances from a location, and the classification and grouping of algorithms are influenced by the effective distance metric [44].

Finally, “IDW algorithm” was used to create clay percentage, silt percentage, sand percentage, pH, carbon, and nitrogen maps in ArcGIS 10.8.0 (Fig 4E–4J).

### 2.4. Determination of silymarin

To quantify the effective chemicals, samples of pyxidiums were collected while they were still in the physiological ripening stage. The seeds were first removed from the pyxidium, and 5 g of seeds from each treatment was finely ground using a mill, enclosed in filter paper envelopes, and subsequently subjected to Soxhlet extraction.

**Fig 5:**
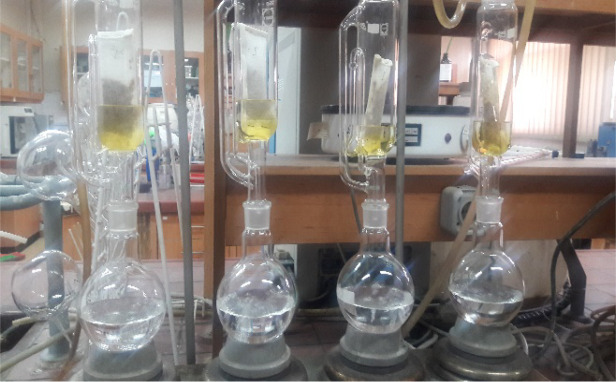
Procedure of measuring secondary metabolites (Silymarin).

Prior to placement in the Soxhlet apparatus, the flasks were weighed and 200 mL of hexane was added to each flask attached to the Soxhlet. The flasks were then heated for 8 h at 65 °C (the boiling point of hexane is 65–70 °C), allowing the oil to separate from the sample and dissolve in hexane. The resulting oil-free powders were oxidized with methanol for 8 h to extract the silymarin. The extracts were subsequently maintained at 50 °C for 5 h to yield a yellow powder after methanol evaporation [45] (Fig 5).

### 2.5. Modeling process

Three algorithms were used to model habitat suitability and changes in the secondary metabolites of *S. marianum*. The RF, BRT, and SVM algorithms were used to model habitat suitability. Similarly, the random forest (RF) algorithm was used to model changes in silymarin levels in Fars Province, Iran.

#### 2.5.1. RF.

Expanding regression tree models are employed within Random Forests (RF), a non-parametric tree-based method that consists of multiple regression and classification trees [46]. The percentage of subsamples, total number of effective estimators for each node, and ideal number of trees are the three characteristics were considered when creating an RF [47]. By utilizing the Bootstrap Aggregation technique, also known as bagging, this method generated an enormous number of noncorrelated trees and determines their average [25]. Large collections of gathered data was split into smaller groups in the decision tree, based on straightforward decision-making criteria in a chain. Groups of sets become increasingly similar to each other with successive classifications [48]. The prediction ability of each tree, as well as the connections between trees, determined RF accuracy [46]. RF widely utilized in machine lerning because it performs well compared with other algorithms for classification and requires fewer testing samples [49,50]. Overfitting is another feature of this categorization technique that removes the necessity for certain processes such as cross-validation [46].

Because the RF technique is non-parametric, it may be used to exploit a variety of explanatory factors and is flexible enough to demonstrate hierarchical relationships among the explanatory variables as well as nonlinear correlations between response factors and explanatory factors [51]. The models in this study were implemented using R version 3.5.3 software (https://cran-archive.r-project.org/bin/windows/base/old/3.5.3/) and the SDM Package [52]. An RF classifier required two parameters to be configured to function: the number of tree classifications and the input factors used at every node [53].

#### 2.5.2. BRT.

The BRT is especially recommended when providing an understanding of ecosystem dynamics, which is of equal importance to model precision [9].

Regression trees and boosting algorithms were used in BRT as a potent modeling technique. Boosted Regression Trees (BRT) provide an effective framework for ecologists to examine the relationships between biological processes and predictor variables. This methodology adeptly accommodates a diverse array of input data types and distributions, as well as efficiently manages missing or erroneous data [54]. The hierarchical architecture of decision trees intrinsic to BRT inherently models interactions among various variables, thereby obviating the need to assume their independence. These capabilities, along with the ability to handle heterogeneous input data, render BRT a highly efficient instrument for ecological research [55,56].

The SDM Package [52], in R version 3.5.3 (https://cran.r-project.org/web/packages/gbm/index.html) was used to operate the BRT models [57]. Because of the assignment of the response variable, the Gaussian was used as the error framework for the loss function in the BRT analysis [58]. The following variables also affected the BRT model fit: (1) cross-validation specifies the number of times the information is randomly split for model fitting and validation, (2) the bagging portion sets the proportion of the findings used to select variables, (3) tree variety controls the level of connections in the BRT, and (4) the learning rate establishes the value of each tree in the growth model [54].

#### 2.5.3. SVM.

Initially, by Cortes and Vapnik [59], “SVM” is supervised learning techniques with corresponding learning algorithms that examine and identify patterns in both input and output data, much like “ANN.” SVM has shown promise as a replacement for determinism modeling and estimating techniques in recent years, and its use warrants further investigation. However, this can occasionally result in random initialization of undeveloped networks and change the stopping criteria when the model parameters are optimized [60]. Such issues and restrictions are missing from SVM-based approaches, which also have a straightforward theoretical foundation and are dependable instruments for modeling and engineering [61]. Model parameters such as the number of nodes and hidden layers do not need to be changed for SVM training techniques, which converge to both local and global optima faster than ANN [59].

The foundation of SVM is the structural risk minimization concept [62]. Among the most advanced non-parametric supervised classification methods currently available, can be configured in a variety of ways based on the kernel function used to create the transform function that converts the input space into the output space. Several functions are commonly employed as kernel functions in SVM, such as the “linear”, “polynomial”, “radial basis function (RBF)”, and “multilayer perceptron” [63]. Essential computations are performed immediately in an input space using kernels [64]. The fact that it is often perceived as a linear method in high-dimensional feature spaces does not necessarily imply that the I/O mapping problem involves high-dimensional features. The models in this study were implemented using “R version 3.5.3” software (version 3.5.3; https://cran.r-project.org/web/packages/gbm/index.html) and SDM Package [52].

### 2.6. Determining the best model

In this research, an ROC curve was used to assess the models and to determine the best model. The Y and X axes in the “ROC curve” are the “true positive rate” and “false positive rate,” respectively; Therefore, if a curve is drawn to the left, the model provides better evaluation. Likewie, if the area under the curve is bigger, it indicates that the model has a better level of accuracy [65,66]. Several studies have confirmed the accuracy of the “ROC curve” [67–70]. The area under the curve shows the accuracy of the models as follows: 0.5–0.6 (poor), 0.6–0.7 (moderate), 0.7–0.8 (good), 0.8–0.9 (very good), and 0.9–1 (excellent) [71].

## 3. Results

### 3.1. Examining the co-linear effect of factors

In this study, 14 factors that influence the growth and development of *S. marianum* medicinal plants were used. Tolerance indices (TOL) and variance inflation factors (VIF) were used to assess collinearity. If TOL < 0.1 and VIF > 5 were fulfilled, collinearity existed between variables [72]. The results of this study showed no collinearity between the independent factors (Table 1).

**Table 1:** The co-linear of factors by TOl and VIF.

Factors	TOL	VIF
Elevation (m)	0.42	2.35
Distance from rivers (m)	0.95	1.05
Distance from roads (m)	0.85	1.18
Sand percent	0.26	3.92
Silt percent	0.73	1.37
Clay percent	0.77	1.29
Carbon percent	0.83	1.19
Nitrogen percent	0.85	1.17
pH	0.66	1.51
Slope degree	0.65	1.49
Slope aspect	0.91	1.09
Mean annual rainfall (mm)	0.52	1.91
Mean annual temperature (°C)	0.32	3.15
Plan curvature (1/100)	0.96	1.03

### 3.2. Quantitative modeling

#### 3.2.1. Habitat suitability by RF.

**Fig 6:**
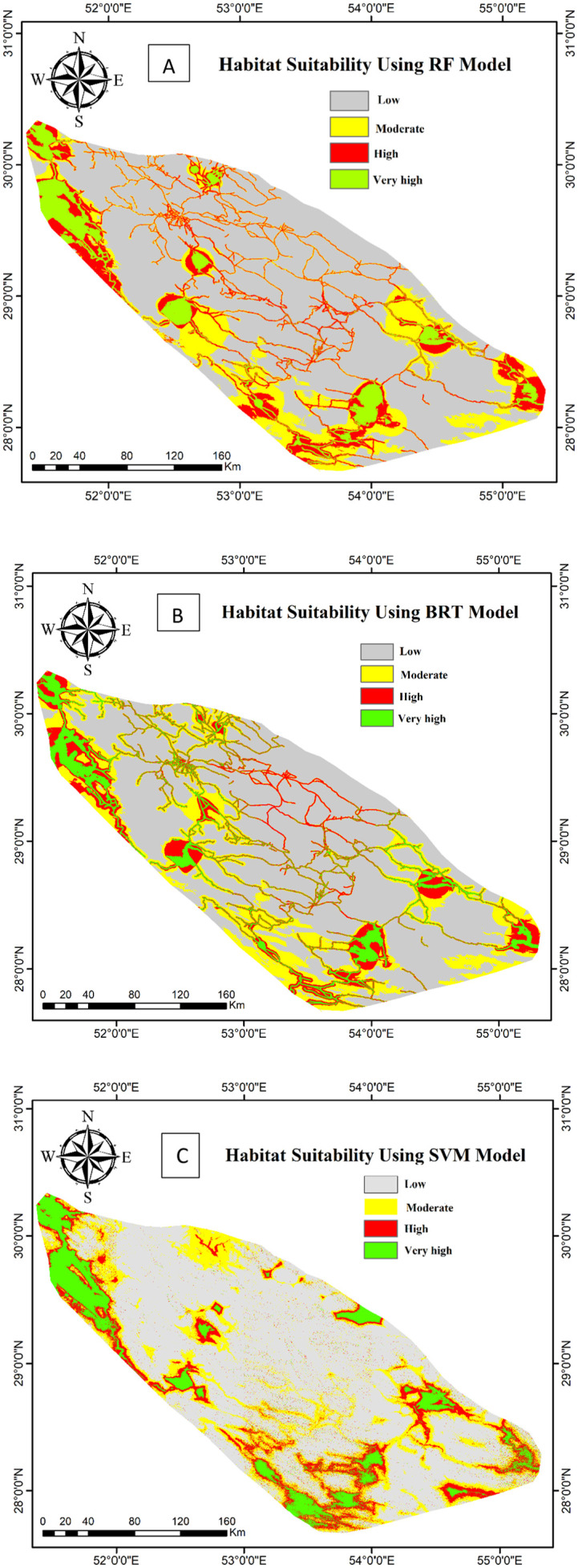
Habitat suitability of *Silybum marianum* using RF(A), BRT (B), and (C) models and silymarin.

The results of random forest model are shown in Fig 6A. The habitat suitability map demonstrated that the distribution of *S. marianum* in the Fars Province was not consistent. For instance, the northwest, west, and south of Fars had more favorable habitats for *S. marianum* (Fig 6A). The habitat suitability map was divided into four classes (low, moderate, high, and very high) using the natural break method [73]. As shown in Table 2, the study area exhibited varying levels of habitat suitability ranging from low to very high.

**Table 2:** Distribution percentage of habitat suitability of *Silybum marianum* in Fars province.

	BRT	RF	SVM
Low	58.99%	61.63%	59.94%
Moderate	20.14%	17.94%	20.37%
High	11.33%	13.80%	10.28%
Very High	9.54%	7.63%	9.41%

#### 3.2.2. Habitat suitability by BRT.

Based on the BRT algorithm, *S. marianum* had greater habitat suitability in the northwest, west, and south of the Fars Province (Fig 6B). As shown in Table 2, habitat suitability was allocated to the low, moderate, high, and very high classes, respectively. As a result, the proportion of each class varies throughout the study region, with the “very high” class having the lowest proportion and the low class having the highest proportion.

#### 3.2.3. Habitat suitability by SVM.

The SVM had approximately the same results as those of RF and BRT. However, the SVM model has a very small difference from the RF and BRT models; therefore, in addition to the northwestern, western, and southern regions, parts of the eastern regions of the Fars Province also have habitat suitability for *S. marianum*. Table 2 presents the habitat suitability across different classes, ranging from low to very high.

### 3.3. Modeling the quality change of silymarin

**Fig 7:**
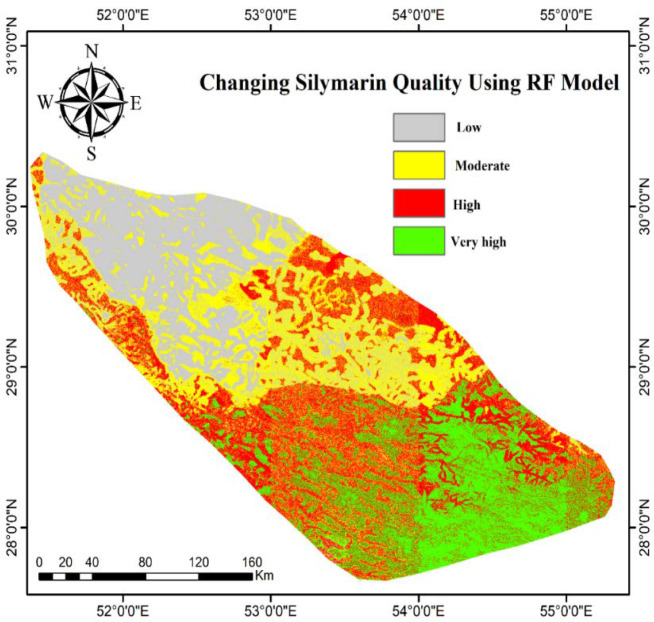
Modeling of changing silymarin quality by RF.

In this study, the “ RF” algorithm was used to model the quality change of silymarin in the Fars Province. These results demonstrate that the concentration of silymarin varied among *S. marianum* from different geographic locations. Similarly*, S. marianum* in the southern region of the Fars Province had greater silymarin concentrations. In addition, *S. marianum* had favorable silymarin concentrations in the central, western, and eastern regions of Fars Province. However, *S. marianum* was significantly decreased in the north (Fig 7). The silymarin ratio of *S. marianum* was categorized as very high, high, moderate, and low (Table 3).

**Table 3:** Percentage change in quality of silymarin in Fars province.

	RF
Low	23.53%
Moderate	27.92%
High	27.15%
Very High	21.40%

### 3.4. Evaluation of classification algorithms

The ROC curve and area under the curve (AUC) were used to assess quantitative models. SVM, BRT, and RF had accuracies of 0.96, 0.98, and 0.99, respectively. These results showed that the habitat suitability of *S*. *marianum* could be accurately predicted using the three aforementioned models (Table 4). The RF model predicted changes in silymarin quality with an acceptable value (RMSE = 0.013), indicating excellent accuracy due to its low RMSE value.

**Table 4:** Choosing the best model by AUC.

Test result variable(s)	Area	Std. error	Asymptotic Sig.	Asymptotic 95% confidence interval	
				Lower bound	Upper bound
BRT	0.98	0.01	0.00	0.96	1.00
RF	0.99	0.01	0.00	0.98	1.00
SVM	0.96	0.01	0.00	0.93	0.99

### 3.5. Determining the importance of the factors

**Fig 8:**
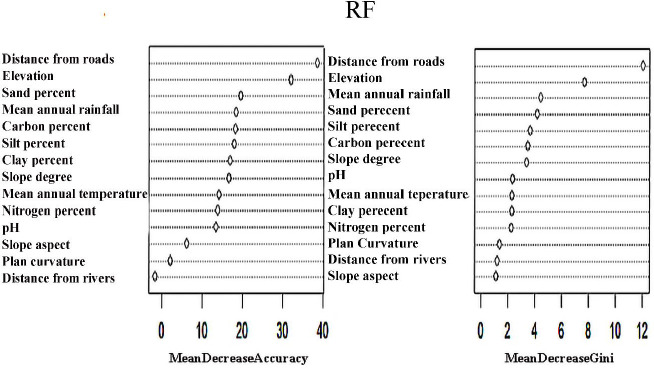
Determining the importance of factors in quantitative modeling by the RF algorithm.

**Fig 9:**
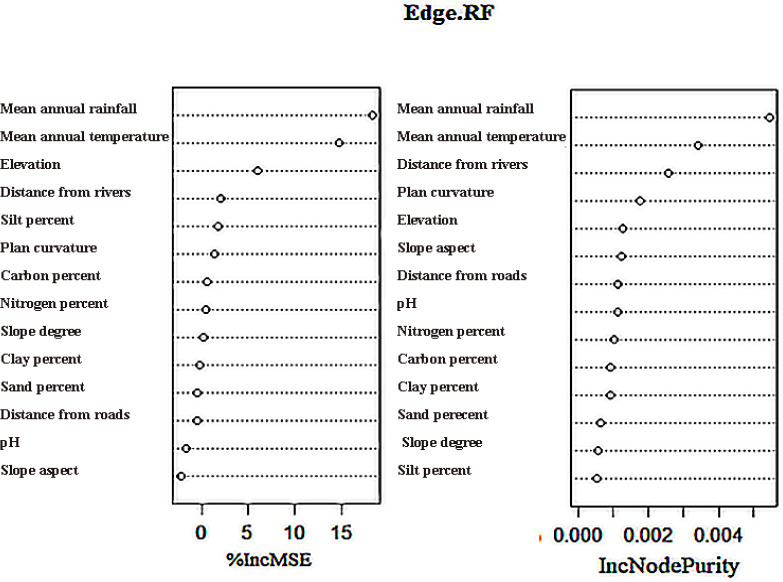
Determining the importance of factors in qualitative modeling by the RF algorithm.

The RF method was used to identify the most significant ecological factors that affect habitat suitability. Distance from roads, elevation, and mean annual rainfall had the greatest effects on habitat suitability and silymarin concentrations in *S. marianum.* However, plan curvature, slope aspect, and distance from the rivers had the lowest impacts (Fig 8). Considering that *S. marianum* in Fars Province often grows alongside roads, the findings regarding the significance of these factors are logical. Similarly, the random forest algorithm results about the importance of factors in the qualitative modeling process showed that the mean annual rainfall, mean annual temperature, and elevation were prominent factors in silymarin accumulation (Fig 9).

## 4. Discussion

Habitat development [74], habitat conservation [75], and invasive species control [76] have all utilized the habitat suitability modeling framework, leveraging the machine learning techniques introduced in this study. This approach is not affected by the geographic location, scale resolution, or plant species distribution. These characteristics may have important ecological applications in the management and conservation of medicinal plants in the future.

Habitat fragmentation poses a threat to the biodiversity. Thus, the primary goal of future conservation programs is to preserve and restore habitat ecosystems [77]. Enforcing beneficial activities requires evaluation of the effectiveness of natural habitats and mapping of their areas. When assessing habitat suitability, the multicollinearity of efficient variables as negative parameters frequently increases the total amount of noise in all the models [78]. However, in this study, none of the environmental, climatic, or soil condition variables included as conditioning elements in the *S. marianum* “habitat suitability model” showed signs of multicollinearity.

Habitat suitability modelingpredicts the number of plant species using important environmental factors [79]. In addition, it is possible to forecast habitats through habitat suitability modeling [80]. Habitat suitability modeling is used for most ecological events to prevent the extinction of plants and animals through accurate predictions [81]. For example, various investigations have utilized habitat suitability modeling to assess the suitability of habitats for various species such as *Panthera tigris,* and *Melursus ursinus* [82,83]. In general, the use of spatial modeling to predict natural events is suggested for the following reasons:(1) protecting the ecosystem, (2) controlling invasive species, (3) preventing the extinction of endangered species, and (4) assisting in managing endangered plant species [84,85]. However, few studies have investigated the qualitative characteristics of these medicinal plants. The Willingness To Pay (W.T.P.) of the local population for the preservation of the Seine Estuary Wetlands, a significant and endangered biological region in Northern France, was determined using a continual valuation survey. In this study, a random forest was used for qualitative modeling [86].

The RF, BRT, and SVM are frequently used [87,88]. Consequently, RF allows for a rapid and simple evaluation of both the current and prospective occurrences of a species [89]. Mollalo et al. [28], proposed that BRT and SVM classifiers are useful and low-cost methods for determining the habitat suitability of a species when combined with GIS and remote sensing data.

The RF model is the most effective and powerful model for determining the habitat suitability [90]. Accordingly, earlier research contrasting the RF and SVM techniques revealed that both models had the highest overall accuracy in terms of forecasting the appropriateness of a given environment [91]. Compared to the CART and GLM models, the BRT model has been shown to be the most sustainable model [92,93]. Massada et al. [94], reported that the BRT and RF models can be used to simulate the occurrence of fires in aforests and rangelands. The number of trees can affect the quality of regression-based models such as BRT and RF [95].

In addition, the models that required a training sample, RF, BRT, and SVM, were optimized in the shortest amount of time; hence, separate training pattern optimization was required for the proximity, density, and inhomogeneity variables. Muñoz-Mas et al. [96], suggested that optimizing training data may enhance algorithmic outcomes. According to Mollalo et al. [28], AUC-ROC is a crucial threshold for associated indices in the presence and absence models of classification (RF, BRT, and SVM) that evaluate how well a model can differentiate between presence and absence. Thus, the AUC statistically generates a single differentiation measure equivalent to the nonparametric Wilcoxon test for all threshold ranges [97]. The AUC values of the RF (0.99), BRT (0.98), and SVM (0.96) models were all reasonable and appropriate, and evaluation of the models did not show a discernible difference between the algorithms. Previous studies have confirmed a small variance in the AUC values for BRT < SVM < RF [98].

The use of machine learning algorithms, including RF, BRT, and SVM, offers significant advantages in conservation and management planning, particularly for habitat suitability modeling. These models handle complex non-linear relationships between predictor variables and species distribution. For instance, RF is robust to overfitting and can identify key environmental factors that influence species distribution [18]. BRT is effective in combining the strengths of regression and decision trees to achieve high predictive accuracy, whereas SVM is particularly suitable for small and imbalanced datasets, offering flexibility in modeling complex ecological patterns [99]. These techniques, coupled with GIS and remote sensing data, facilitate the creation of spatially explicit maps that are essential for identifying priority conservation zones [100].

Despite their benefits, however, these models have certain limitations. RF and BRT can be computationally demanding, particularly for large datasets, and may lack interpretability compared to traditional statistical approaches [101]. SVM requires careful parameter optimization, and its performance can be degraded using very large datasets. Another common challenge is the reliance of these models on high-quality input data; inaccuracies in environmental or spatial datasets can propagate errors in final predictions. Furthermore, the transferability of models across regions or under future climate scenarios requires validation to ensure their reliability [18].

By understanding these advantages and limitations, researchers can make informed decisions regarding appropriate modeling techniques for specific ecological and conservation objectives, ensuring robust and actionable outcomes for habitat management.

The geographic distribution of species within their habitats is significantly influenced by environmental conditions in significant ways [102]. A substantial correlation between the size of the training dataset and eco-geographic variables (EGV) has been reported when predicting habitat suitability using RF, BRT, and SVM models [102–104]. Overall, it has been discovered that topography, temperature, and precipitation have greater detrimental effects on species dispersion [105]. In their native environments, *S. marianum* varies according to ecological and climatic conditioning variables [106]. Previous studies have also reported this phenomenon in other plants. For example, according to Kunwar et al. [107], the primary determinants of *Juniperus occidentalis* abundance and distribution include long-term temperature variations, the quantity and distribution of rainfall, and the size and length of fire outbreaks. Temperature, precipitation, and altitude are the most important variables that influence *J. drupacea* are temperature, precipitation, and altitude [108]. Furthermore, elevation and precipitation have an impact on *J. excelsa* distribution patterns in Lebanon [109]. However, the findings of the factor importance analysis showed that the three most crucial variables in *S. marianum* habitat suitability modeling were elevation, mean annual rainfall, and distance from roads. According to these findings, *S. marianum* accumulated to a greater extent in regions close to roads. On the other hand, the variables that showed the greatest influence on the accumulation of silymarin in *S. marianum* were elevation, mean annual temperature, and mean annual rainfall.

The results showed that there was a higher *S. marianum* population close to highways. Additional studies have shown that roads can positively affect the distribution and survival of plant species [110]. Human disturbance is one of the primary factors affecting habitat alteration in plants is human disturbance [111]. Zhang and Ma [112], emphasized the impact of highways on the diversity of plant habitats. They found that one of the key variables influencing species richness is the presence of roads [113]. Roads can change species assemblages by affecting the seed dispersal.

In the present study, the random forest algorithm’s categorization ranked elevation as the second most influential factor. These results indicated that low altitudes often exhibit the highest habitat suitability for *S. marianum*. Tiwari et al. [114], reported that elevational factors affected plant distribution. For example, plants usually grow better at lower elevations than at **upper** elevations. Vegetation is widely dispersed at altitude above 1000 m [112]. Topography, particularly elevation, may affect plant distribution [115]. Thus, elevation may affect the surface water flow, erosion, plant surface penetration, soil formation over time [116], climate [117], and seed dispersion [118].

These findings revealed that the density of *S. marianum* is directly influenced by decreasing rainfall. According to Naghipour Borj et al. [119], precipitation is one of the factors influencing the spread of medicinal plants throughout temperate and semiarid regions. Future and current climate changes are expected to disrupt grasslands and agriculture. Temperature and precipitation play a crucial role in the distribution of medicinal plants. It has been demonstrated that ambient precipitation is a significant indicator of the distribution of medicinal plants within a given region [120]. Kefalew et al. [121], found that “temperature and precipitation” in semi-arid regions can affect the distribution of medicinal plants. Additionally, Dong et al. [122], reported that an increase in temperature caused by climate change leads to an increase in secondary metabolites. These metabolites defend against high temperatures by increasing lignin, promoting sclerophilic tissues, and synthesizing secondary compounds such as phenolic compounds and sesquiterpenes. These findings obtained support this argument as the concentration of silymarin notably increased in the southern regions characterized by warmer temperatures.

Recent studies have demonstrated the importance of qualitative modeling and advocated its application [123,124]. Assessing the quality of *S. marianum* across different regions of Fars Province is crucial because of its silymarin content, a significant compound utilized in treating conditions such as fatty liver and chronic inflammatory liver diseases such as liver cirrhosis. Hence, this study aimed to delineate variations in silymarin content across the Fars Province using a random forest algorithm. These findings suggest that the RF model demonstrated outstanding accuracy. Zhang and Wang [124], suggested that the random forest approach could be applied to qualitative modeling of medicinal plants.

Determining the habitat suitability and quality of *S*. *marianum* is crucial, because it is generally recognized as an important medicinal plant. One noteworthy aspect of this study was the comparison between the maps of habitat suitability and changes in silymarin quality. This demonstrates that while *S*. *marianum* habitats may be identified in various locations, the concentration of silymarin is not consistently high across these areas. This study demonstrated that silymarin content tends to be higher in plants found in the southern part of Fars Province with warmer climate. However, because *S. marianum* is a medicinal plant that grows at cooler temperatures, the western and northern locations are more desirable habitats. Nevertheless, it is important to note that elevated concentrations of silymarin were not expected in colder regions.

Distance from roads is the most crucial aspect of quantitative modeling. It appears that the proliferation of roads has led to the dispersal of *S. marianum* seeds at various locations. On the other hand, the “mean annual rainfall and mean annual temperature” had the greatest effect on silymarin quality. This study Showed that high temperatures and low rainfall increased silymarin levels. The results of this study may be used to expand the habitat of *S*. *marianum* to southern regions, thereby producing more silymarin.

## 5. Conclusions

This study highlights the critical findings regarding habitat suitability and silymarin accumulation in *S*. *marianum*. Key environmental factors influencing habitat suitability include distance from roads, elevation, mean annual rainfall, and soil properties. Additionally, RF effectively identified these factors as the primary contributors to the spatial distribution of *S*. *marianum* and accumulation of silymarin, a valuable medicinal compound. This Study showed that high temperatures and low rainfall increased silymarin levels. The results of this study may be used to expand the habitat of *S*. *marianum* to southern regions, thereby producing more silymarin. These findings provide a robust framework for understanding the ecological preferences of *S*. *marianum* and optimizing its cultivation and management for pharmaceutical applications. By identifying the most influential environmental variables, this research advances the potential for the sustainable utilization of this species, enhancing both its conservation and use as a medicinal resource.

## Supporting information

S1 File Hojati data 2. (XLSX)
